# Incidental Finding of Coronary Artery Dilatation in Children With History of COVID-19 Having Minimal or No Symptoms: Raising Red Flag

**DOI:** 10.7759/cureus.24348

**Published:** 2022-04-21

**Authors:** Adeel Khalid, Najma Patel, Zain Yusuf Ally, Shaista Ehsan

**Affiliations:** 1 Pediatric Cardiology, Ziauddin University, Karachi, PAK; 2 Pediatric Medicine, Ziauddin University, Karachi, PAK

**Keywords:** left main coronary artery aneurysm, palpitation, intermittent chest pain, kawasaki-like, multisystem inflammatory syndrome in children (mis-c), covid 19, coronary artery dilatation

## Abstract

Coronary artery dilatation has been observed with coronavirus disease 2019 (COVID-19)-associated multisystem inflammatory syndrome in children (MIS-C), which is more common in those with Kawasaki-like disease. MIS-C is a clinical syndrome in children and adolescents; its signs and symptoms, as well as cardiac manifestations, are similar to Kawasaki diseases, such as coronary artery dilation, coronary aneurysms, and ventricular dysfunction. The occurrence of coronary artery dilatation in asymptomatic pediatric patients following COVID-19 infection has not been well documented in the literature.

Thus, in this article, we present four cases of coronary artery dilation in children with a past history of COVID-19 infection who had very few or no symptoms and were referred to us for vague chest pain and palpitation. As a result, a high index of suspicion is required, and any patient complaining of chest pain and palpitation with a history of COVID-19 exposure should not be ignored and be given proper coronary artery evaluation. This article also raises the question of whether every child infected with COVID-19 should have an echocardiogram.

## Introduction

Coronary artery dilatation has been reported in children with the multisystem inflammatory syndrome (MIS-C), both with and without overlap with Kawasaki disease (KD). However, it is reportedly more common in children who have KD. MIS-C is a clinical syndrome in children and adolescents, which was first identified in association with a high prevalence of COVID-19 infection. MIS-C illness is often distinguished by persistent fever, laboratory markers of inflammation, and evidence of single or multiorgan dysfunction. It may have symptoms of Kawasaki syndrome (conjunctival and mucosal injection, rash, swelling of the hands and feet, coronary artery dilation) or toxic shock syndrome (erythroderma, renal involvement, and hypotension). Cardiac manifestations, such as ventricular dysfunction, coronary artery dilation and aneurysms, arrhythmia, and conduction abnormalities are common in MIS-C [1].

According to a study described in COVID-19-associated multisystem inflammatory syndrome in 614 children with and without overlap with KD, coronary artery dilatation was seen in patients with overlap KD with a significant p-value (13.4% vs. 6.8%, p = 0.007), while myocarditis was significantly more common in patients without overlap with KD features (2.6% vs. 7.4%, p = 0.009) [2].

Coronary artery dilatation has not been reported in children who have a history of COVID-19 infection but have very few or no symptoms. In this article, we present four cases of incidental coronary artery dilation in children who had a history of COVID-19 infection but had very few or no symptoms and were referred for vague chest pain.

## Case presentation

Patient 1

A nine-year-old boy (weight: 54 kg, BSA: 1.47 m^2^) was referred to our Pediatric Cardiology Clinic with complaints of palpitation and chest pain for one week. The exertion caused chest pain that lasted three to five minutes. He had a history of febrile illness nine months prior when his parents tested positive for COVID-19 via polymerase chain reaction (PCR) test. He only had a low-grade fever and a mild cough for four days before his COVID-19 PCR test came back positive.

At the time, he did not have a sore throat, rash, edema, or conjunctivitis. He did not require hospitalization, and his condition improved after he was given an antipyretic and oral antibiotic medication. He recovered from that illness and has remained stable since then.

**Figure 1:**
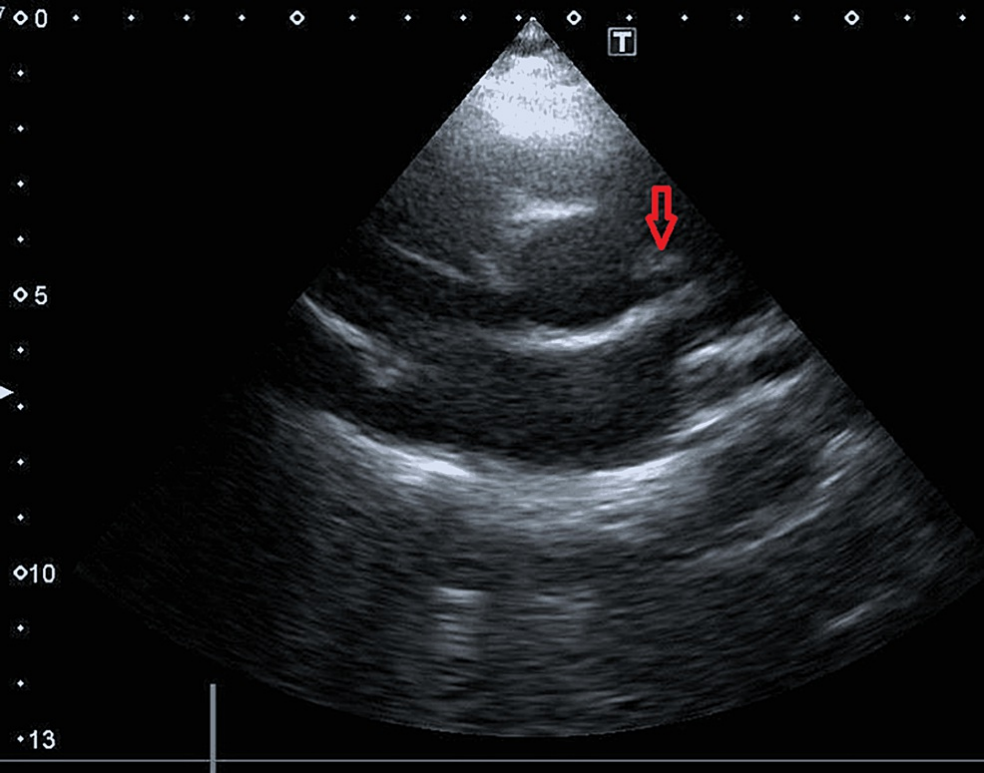
Enlarged left coronary artery in short axis view shown with red arrow

**Figure 2:**
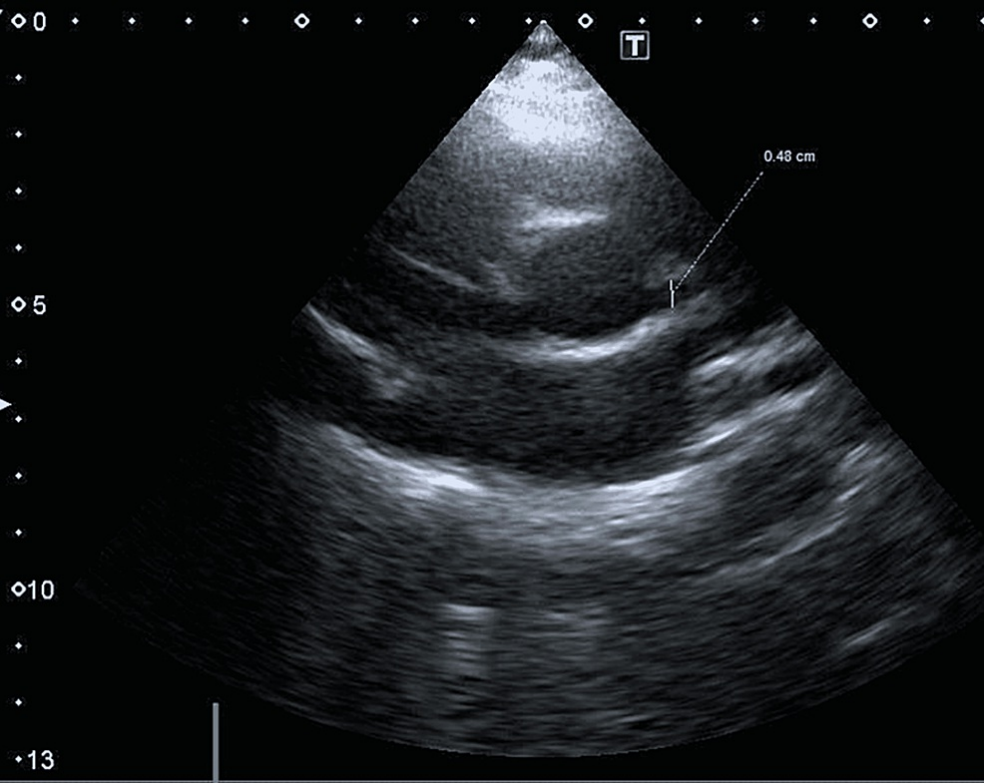
Short axis view showing dilated proximal left coronary artery, measuring 4.8 mm in diameter

His electrocardiogram was deemed unremarkable. Echocardiography documented a structurally normal heart with normal systolic and diastolic functions. The left coronary artery was dilated proximally and fusiform in shape, measuring 4.8 mm (Z-score 2.95), tapering to 3 mm distally with perivascular brightness (Figures 1, 2). The right coronary artery appeared to be normal.

The patient was physically active with no activity limitations. He was advised to take an antiplatelet dose of aspirin. His heart rate, blood pressure, fasting blood sugar level, and thyroid profile were all within normal limits.

**Figure 3:**
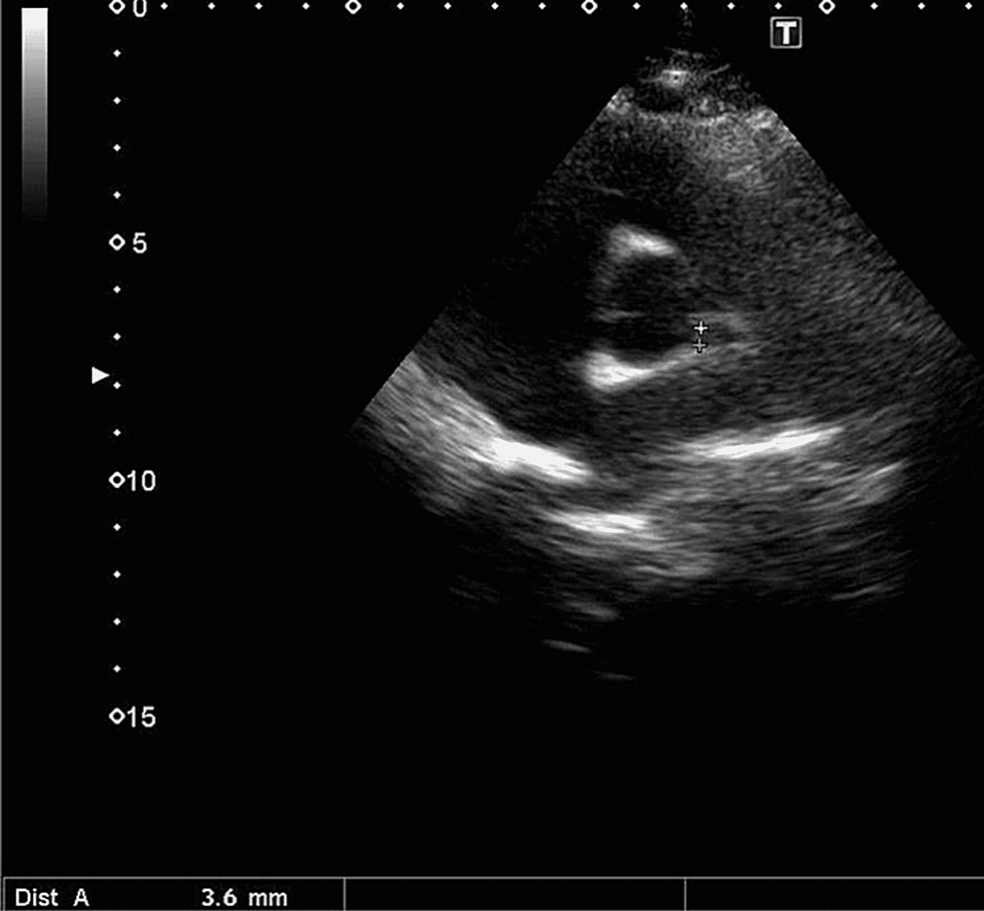
Left coronary artery measuring 3.6 mm

It was suggested that strenuous activity be restricted. In a follow-up echocardiography after six months, his left coronary artery Z-score improved from 2.95 to 0.13 (Figure 3).

Patient 2

**Figure 4:**
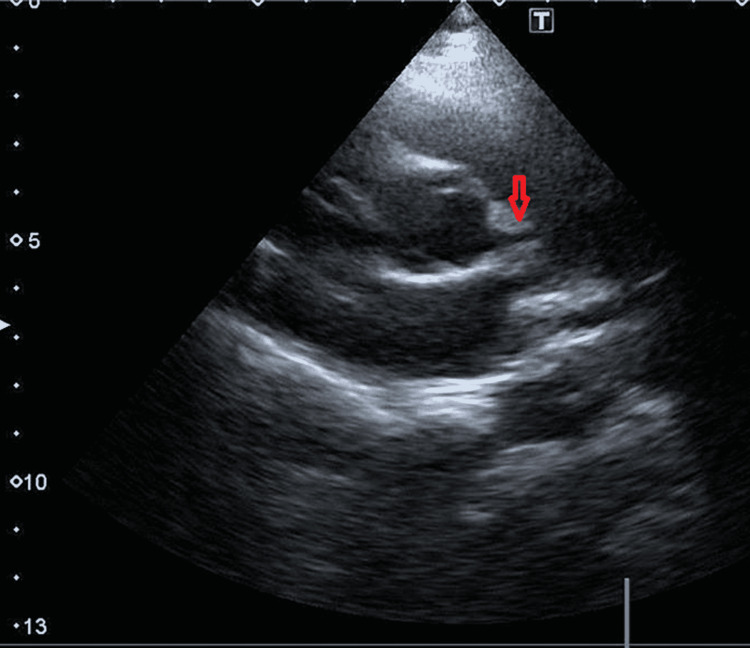
Enlarged left coronary artery shown with red arrow

**Figure 5:**
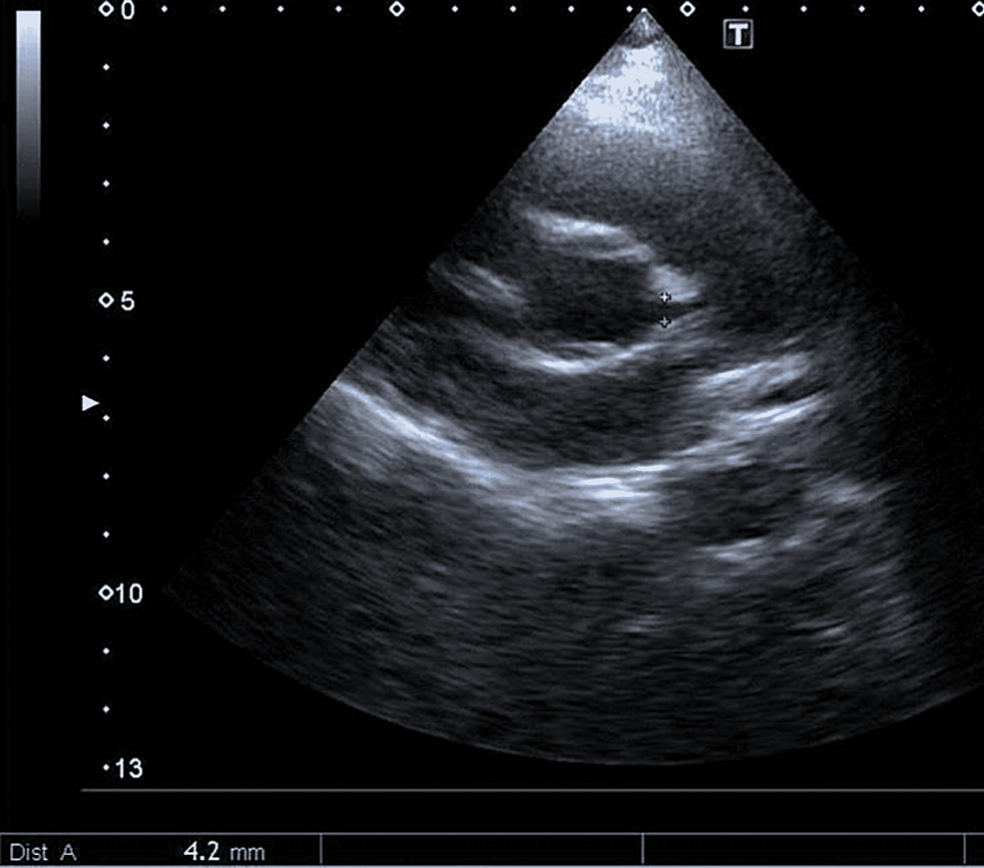
Short axis view showing dilated proximal left coronary artery, measuring 4.2 mm in diameter, with some distal tapering

Another four-year-old boy (weight: 18 kg, BSA: 0.71 m^2^) came to our clinic complaining of chest pain on exertion for five days. His older brother and mother were both positive for COVID-19 two months ago; however, the patient was asymptomatic. After his screening, his COVID-19 PCR test also came back positive. His electrocardiogram was unremarkable. An echocardiogram revealed a 4.2 mm proximal dilated left coronary artery (Z-score 4.86) (Figures 4, 5). The right coronary artery was normal.

He was also advised to take aspirin. After three months, in a follow-up echocardiography, his left coronary artery measured 3.5 mm (Z-score 3.08).

Patient 3

**Figure 6:**
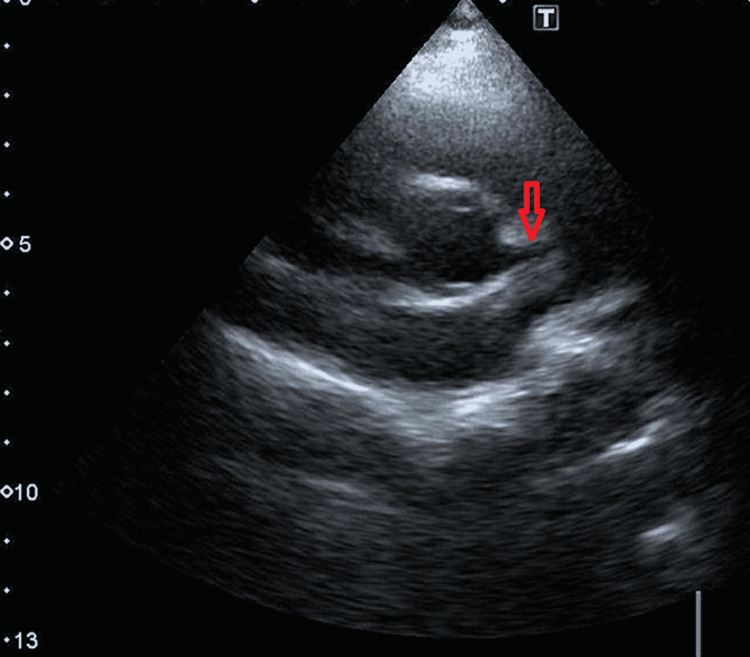
Enlarged left coronary artery shown with red arrow

**Figure 7:**
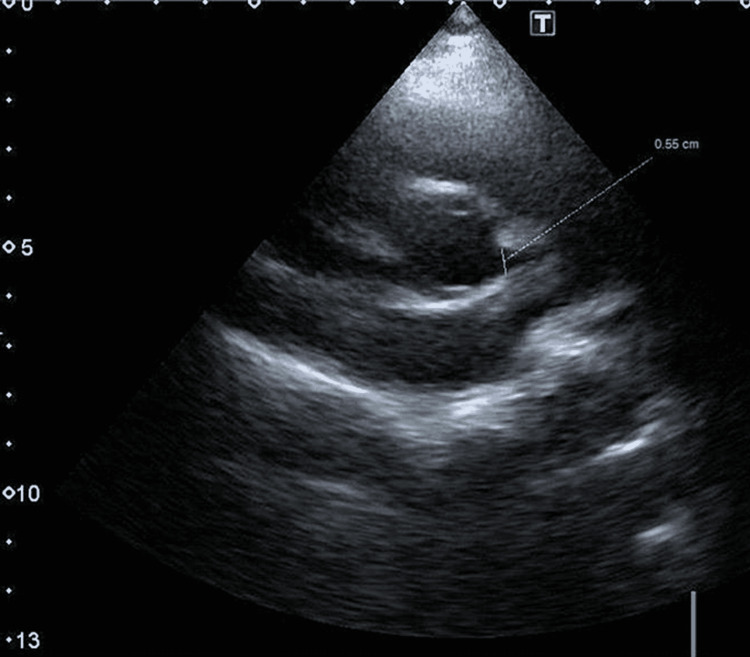
Short axis view showing dilated proximal left coronary artery, measuring 5.5 mm in diameter

The third case was an 11-year-old kid (weight: 58 kg, BSA: 1.54 m^2^), who presented in our clinic with a history of chest pain for one week, more on exertion; however, parents did not take notice of this. However, two days before consultation, the child experienced severe chest pain while at school. His parents were contacted, and he was sent home with them. The child had no febrile illness; however, after a few days, his father developed fever, sore throat, and myalgia, for which he was advised to take a COVID-19 PCR test, which came back positive. As a result, we believe the child may have acquired COVID-19 infection, although his PCR test was not performed at the time of presentation. His echocardiography also showed a dilated left coronary artery proximally, measuring 5.5 mm (Z-score 4.31) (Figures 6, 7) with perivascular thickening.

Patient 4

The fourth case was a six-year-old boy (weight: 19 kg, BSA: 0.76 m^2^), who presented in our clinic with a vague complaint of palpitation on and off for two weeks. When asked about his medical history, he mentioned having fever and diarrhea three months ago. His mother was COVID-19 positive at the time, and his COVID-19 antibodies were negative. His electrocardiogram was normal, but echocardiography revealed a proximal dilated left coronary artery measuring 3.6 mm (Z-score 3.07) with perivascular thickening of 2.2 mm in the right coronary artery (Z-score 0.25). He was also advised to take an antiplatelet dose of aspirin and was given a proper follow-up schedule. All of the above-mentioned cases are summarized in Table 1.

**Table 1:** Characteristics of patients with coronary artery dilatation without MIS-C MIS-C - multisystem inflammatory syndrome in children

	PATIENT 1	PATIENT 2	PATIENT 3	PATIENT 4
AGE (years)	9	4	11	6
WEIGHT (kg)	54	18	58	19
SEX	MALE	MALE	MALE	MALE
Evidence COVID 19 STATUS	PCR Positive	PCR Positive	Test not done but history of COVID in family	COVID antibodies negative H/O COVID in family
PRESENTATION	Palpitation and chest pain	Chest pain	Chest pain	Palpitation
CORONARY ARTERY	Left coronary artery	Left coronary artery	Left coronary artery	Left coronary artery
CORONARY ARTERY (C.A) Z SCORE	2.94	4.86	4.34	3.07

## Discussion

Cardiac effects in both adults [3] and children [4] with COVID-19 infection have been described, including significant cardiac involvement in pediatric patients who develop MIS-C. Various case studies reported coronary artery dilatation or aneurysms in 6%-24% of critically ill MIS-C patients [5]. COVID-19-related cardiac injury is thought to be caused by a variety of mechanisms, including cardiac myocyte injury caused by an acute and dysregulated inflammatory response related to cytokine storm, microvascular dysfunction, and viral invasion of cardiomyocytes causing cellular damage, and ischemic injury [6].

In the adult population, it is recommended that patients with mild to severe COVID-19 disease undergo cardiac evaluation before returning to strenuous physical activity [7]. MIS-C or moderate to severe disease, on the other hand, necessitates cardiac evaluation in the pediatric population. In all four patients, symptoms were noted to be very mild, i.e., fever for two to three days, and one patient was found to have been positive for COVID-19 during screening and had no symptoms. Presenting complaints were very nonspecific as vague chest pain and complaints of palpitation are very common and have no pathological basis. All of them had left coronary artery dilation, with a mean Z-score of 3.8 [8]. A coronary artery Z-score of >2 is considered significant. Table 1 summarizes the findings.

There is only one case report of coronary artery dilatation in an asymptomatic patient with COVID-19, as described by Gerber et al. in 2021 [9] in which there was just borderline uniform dilatation of the coronary artery. In all four of our cases, the coronary artery had aneurysmal proximal dilation with distal tapering.

As a result, any patient who complains of chest pain and palpitation and has a history of COVID-19 exposure should not be ignored and should have their coronary arteries evaluated. In contrast to KD, which is common in children under the age of five, the age group in all four described cases was older [10].

This warrants assessment for coronary involvement in children who were found to have COVID-19 even with mild symptoms or no symptoms. However, this will place a significant strain on healthcare facilities. Second, it has a psychological impact on both parents and children. All four parents were unwilling to accept it, necessitating lengthy and multiple sessions of counseling, and we were in constant contact with them to ensure their adherence to antiplatelet therapy. Their progression is similar to that of KD. We also examined a large number of patients with MIS-C and myocarditis, both with and without COVID-19 infection; however, coronaries were found to be normal in these patients.

After having these findings, we started to look at coronaries carefully in all patients who had echocardiography in our hospital. During the previous year, 10 patients had acute myocarditis, and three had vague chest pain but no history of COVID-19. The coronary arteries in each of them were normal. However, based on these cases, we have begun a study in children with proven COVID-19 infection.

## Conclusions

There must be a high index of suspicion of coronary artery dilatation in patients who had a history of COVID-19 infection. Cases mentioned in our study highlight the risk for cardiac involvement and development of coronary aneurysms in patients with minimal to no symptoms, and all except one presented after a few months of having COVID-19, and all had episodic chest pain. It calls for a retrospective and prospective study in children who are found to be COVID-19 positive, whether symptomatic or not, as well as those who have recently had an infection that requires long-term monitoring.
